# Glioblastoma Molecular Classification Tool Based on mRNA Analysis: From Wet-Lab to Subtype

**DOI:** 10.3390/ijms232415875

**Published:** 2022-12-14

**Authors:** Giedrius Steponaitis, Vytautas Kucinskas, Ieva Golubickaite, Kestutis Skauminas, Ausra Saudargiene

**Affiliations:** 1 Laboratory of Molecular Neurooncology, Neuroscience Institute, Lithuanian University of Health Sciences, Eiveniu Str. 4, LT-50161 Kaunas, Lithuania; 2 Laboratory of Biophysics and Bioinformatics, Neuroscience Institute, Lithuanian University of Health Sciences, Eiveniu Str. 4, LT-50161 Kaunas, Lithuania

**Keywords:** glioblastoma, molecular subtyping, classification tool, biomarkers, mesenchymal, proneural, classical

## Abstract

Most glioblastoma studies incorporate the layer of tumor molecular subtype based on the four-subtype classification system proposed in 2010. Nevertheless, there is no universally recognized and convenient tool for glioblastoma molecular subtyping, and each study applies a different set of markers and/or approaches that cause inconsistencies in data comparability and reproducibility between studies. Thus, this study aimed to create an applicable user-friendly tool for glioblastoma classification, with high accuracy, while using a significantly smaller number of variables. The study incorporated a TCGA microarray, sequencing datasets, and an independent cohort of 56 glioblastomas (LUHS cohort). The models were constructed by applying the Agilent G4502 dataset, and they were tested using the Affymetrix HG-U133a and Illumina Hiseq cohorts, as well as the LUHS cases. Two classification models were constructed by applying a logistic regression classification algorithm, based on the mRNA levels of twenty selected genes. The classifiers were translated to a RT-qPCR assay and validated in an independent cohort of 56 glioblastomas. The classification accuracy of the 20-gene and 5-gene classifiers varied between 90.7–91% and 85.9–87.7%, respectively. With this work, we propose a cost-efficient three-class (classical, mesenchymal, and proneural) tool for glioblastoma molecular classification based on the mRNA analysis of only 5–20 genes, and we provide the basic information for classification performance starting from the wet-lab stage. We hope that the proposed classification tool will enable data comparability between different research groups.

## 1. Introduction

The molecular classification of glioblastoma has received increasing scientific attention over the past 15 years [1,2]. Since glioblastoma is one of the most lethal (with a median survival of 15 months) and incurable human diseases [3,4], different types of diagnostic, prognostic, and therapeutic approaches have to be applied to comprehend these issues. The inter- and intra-tumor heterogeneity of the molecular landscape became one of the most interesting features of GBM that was analyzed by examining different types of molecules. The main purpose was to classify tumors into more homogenous groups with similar behavior with regard to similar response to therapy, comparable course of the disease, probability of tumor relapse, comparable patient survival, etc. One of the first GBM classification studies published in 2006 by Phillips et al. suggested the three-group (proneural—PN, proliferative—Prolif, and mesenchymal—Mes) subtyping model, based on the signature of 35 genes mRNA expression [5]. In 2010, a four-subtype classification model, referred to as proneural—PN, classical—CL, mesenchymal—ME, and neural—NE, which is tightly associated with genomic abnormalities, was proposed by Verhaak et al. based on the mRNA expression of 840 genes [6]. During the past 10 years, dozens of schemes have been suggested for molecular classification of glioblastoma, introducing 3–5 subtypes [7,8,9,10,11,12]. Verhaak classification became the main system that has been widely used to analyze differences in treatment efficiency and resistance, course of the disease, overall patient survival, tumor impurities, immune microenvironment activation level, etc. in relation to GBM subtypes [1,2,9,10,11,12,13,14,15,16,17]. Nevertheless, ambiguous data were published regarding the interrelation of glioblastoma’s clinical and molecular (subtype) features. The inability to accurately determine patient outcomes based on molecular subtypes was the main issue discussed [9,15]. Despite the fact that the GBM subtyping only partially discloses tumor behavior and patient outcome, evidently, different molecular profiles of different GBM subtypes determined scientific interest. Moreover, the different molecular landscape of glioblastomas indicates that tumors are acting through subtype-specific regulatory pathways [18]. Thus, distinct and subtype-specific targets and therapies are needed for disease cure [19]. In addition to the 2016 World Health Organization (WHO) updated guidelines for GBM classification incorporating *IDH* mutation and *MGMT* methylation status [3], the vast majority of GBM studies also analyzed data incorporating the Verhaak proposed subtype. Even though the Verhaak four-group subtype system became the most acceptable, researchers are still applying different methods and markers to assign subtypes to a newly analyzed tumor or glioblastoma-derived cell line. Different assays are used, since scientists are trying to simplify the subtyping process instead of analyzing the expression of hundreds of transcripts.

Despite the fact that many glioblastoma molecular classification models were suggested since the Verhaak classification system was proposed [7,8,10,11,12,20,21,22,23,24], only two glioblastoma classification tools (based on Verhaak four-groups classification) were proposed in 2014 [20] and 2016 [21]. However, during the last several years, few studies have demonstrated that specimens of the neural subtype are samples with a high content of normal tissue, rather than a separate tumor subtype [11,12,25]. Therefore, no clearly defined, accurate, three-group, user-friendly, open-access, and generally accepted tool has been suggested up to this point. In the present work, we developed a three-group classifier tool that enables the assigning of glioblastoma specimens or glioblastoma-derived cell lines to the Verhaak’s proposed subtypes (PN, CL or ME) based on 5–20 gene mRNA levels. In addition to TCGA data analysis, we performed a mRNA expression analysis of 20 selected genes in the cohort of 56 glioblastoma specimens to optimize and validate the methodological part of the subtyping. The developed user-friendly glioblastoma classification tool is available for every scientist, with no bioinformatic background, and enables the classification of glioblastoma in a cost-effective way. The classifier tool is capable of assessing the glioblastoma subtype using 5–20 specified genes’ mRNA data, depending on the desired accuracy.

The GBM classification tool is designed based on machine learning and the data visualization toolkit “Orange”. The subtyping model, data normalization, and the file architecture instructions, as well as general usage instructions, are available to download at: https://github.com/GiSteps/GBM-Molecular-Classifier (accessed on 1 December 2022).

## 2. Results

### 2.1. The Design of Classification-Relevant Markers Selection

**Figure 1:**
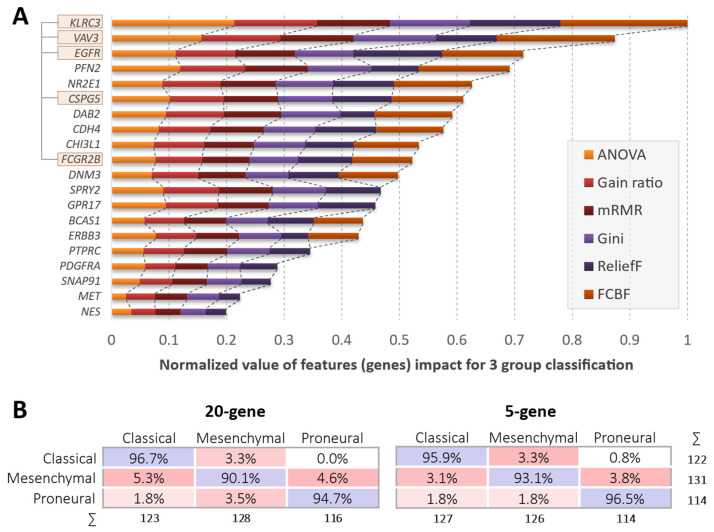
(**A**) Twenty genes were selected for GBM subtype classifier development, which scored the highest cumulative values regarding the feature selection methods. The values of each feature measured by the different methods were normalized to the method values mean. Genes selected for the five-gene classifiers are indicated by pink rectangles. (**B**) The comparison of 20-gene and five-gene classifiers’ classification accuracy per tumor subtype.

Signature–gene selection for GBM subtyping was based on preselected signatures proposed elsewhere [5,6,11,26]. After combining gene lists from four studies with proposed subtyping signatures, a total of 77 unique genes were received. We then overlapped the list between two gene expression array platforms: the Affymetrix HG-U133a and the Agilent G4502 datasets. The expression data of 69 out of 77 primarily selected genes overlapped in all datasets; therefore, 69 overlapping targets were selected for further analysis (see Supplementary Table S1). Since the same specimens were used for mRNA-level analysis using both microarray platforms, we performed correlation analysis on selected targets to evaluate the mRNA-level reproducibility of each target. The targets having a Pearson correlation coefficient lower than 0.65 were eliminated from further study. Fifty-four selected genes were used to rank the top 20 ones for GBM subtyping using a feature selection model applying ANOVA, ReliefF, Gini decrease, gain ratio (GR), fast correlation-based filter (FCBF), and minimum redundancy maximum relevance (mRMR) methods. Next, the lists were overlapped, and 20 genes that scored the highest cumulative values from feature selection methods were selected (see Figure 1A).

### 2.2. Classifier Development

Data from the Agilent G4502 platform were applied for classification model building. The construction of the classifier incorporated only mesenchymal, classical and proneural cases, since recently it was shown that TCGA samples of the neural subtype are samples with a high content of normal tissue, rather than a separate subtype of tumor [12]. After the case filtering described in the methods section and neural subtype elimination, a total of 419 cases were used for model building (MES *n* = 151; CL *n* = 140; and PN *n* = 128). We tested two classification algorithms: logistic regression with LASSO (L1) and support vector machine (SVM) for classifier development to select the most suitable one. Both algorithms revealed highly comparable results applying a 10-fold cross-validation design. We reduced the number of genes to suggest the minimal number of genes that allows tumor classification with an acceptable accuracy. We found that a minimum of five genes is required to receive an overlap with the original classification of at least 85%. First, we selected the first top five ranked genes according to the ANOVA scoring method (Anova was revealed to be the most representative of all methods used, see Figure 1A). Next, we tested the combination of five features to select the most accurate, and we found that the panel of *KLRC3*, *VAV3*, *EGFR*, *CSPG5*, and *FCGR2B* (Figure 1A) revealed the highest classification accuracy (>93%) that was comparable with the 20-gene classifier accuracy (>95%) (see Table 1). The classification accuracy applying the five-gene model revealed even slightly higher accuracy when recognizing the mesenchymal subtype (see Figure 1B). Both algorithms revealed similar results. Nevertheless, the final model is built on the logistic regression classification algorithm, since it showed a more accurate classification during the validation stage (Table 1).

### 2.3. Validation and Testing of Classification Models

**Figure 2:**
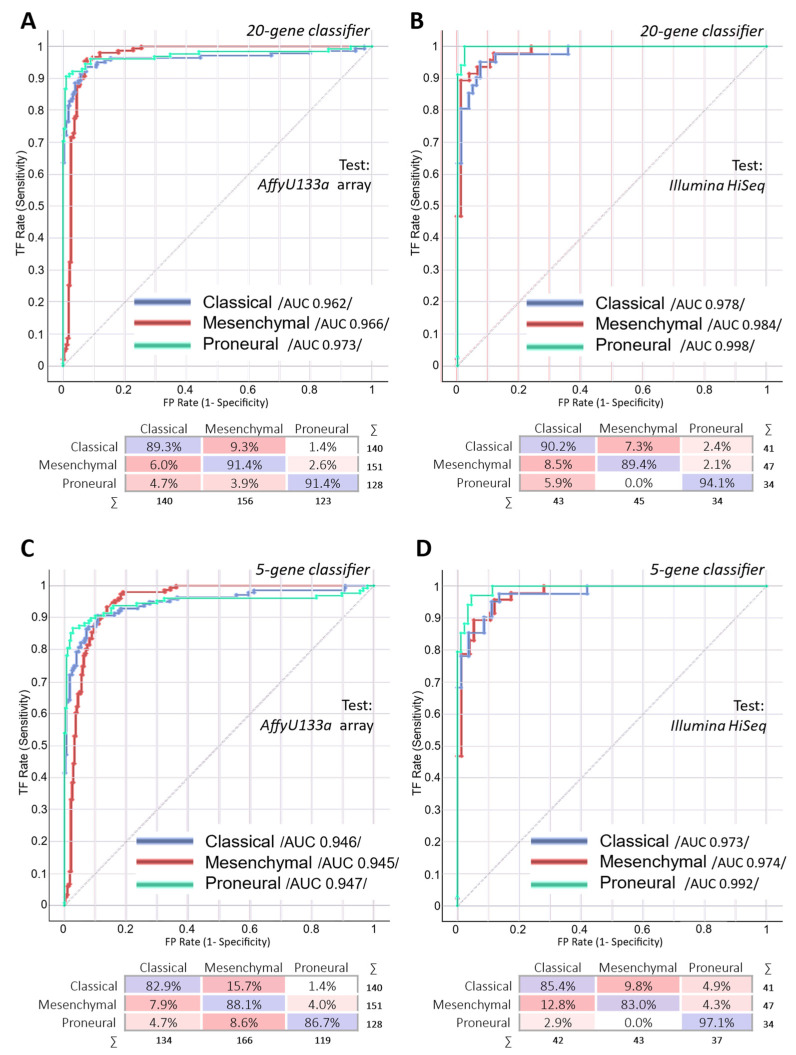
Receiver operating characteristic curves of the 20-gene classifier testing the Affymetrix HT expression array cohort (**A**) and Illumina HiSeq RNA sequencing data cohort (**B**). ROC analysis of the five-gene classifier testing the Affymetrix HT expression array and Illumina HiSeq RNA sequencing data cohorts indicated in parts (**C**) and (**D**), respectively. The matrix at the bottom of each ROC plot specifies the classification accuracy for each subtype.

The classification models were tested on two public datasets generated by a gene expression array (Affymetrix HT) and by RNA sequencing (Illumina HiSeq). The gene expression microarray dataset consisted of 419 (MES *n* = 151; CL *n* = 140; PN *n* = 128) samples, while the RNA sequencing dataset consisted of 122 (MES *n* = 47; CL *n* = 41; PN *n* = 34) cases (neural subtype cases were removed from both datasets). We applied both classification models (20-gene and 5-gene) for testing. As was suspected, the classification accuracy of data generated by different array platforms or even methods was slightly lower, but it was, nevertheless, sufficiently high to be used as a tool. The accuracy of the 20-gene classifier of the Affymetrix HT dataset reached 90.7%, while the Illumina HiSeq dataset revealed 91% of overall accuracy. Five five-gene classifiers revealed 85.9% classification accuracy when testing the Affymetrix HT dataset and 87.7% accuracy when testing the Illumina HiSeq cases (see Figure 2).

**Figure 3:**
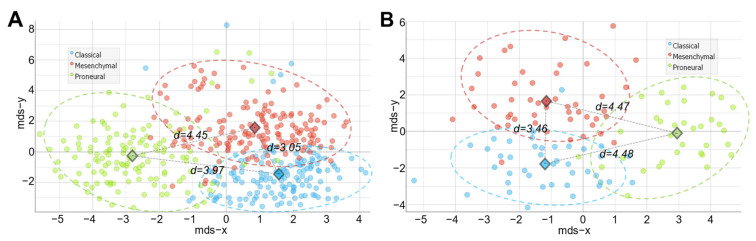
Multidimensional scaling (MDS) analysis applying 20 selected subtyping genes. The distance visualization of the Affymetrix HT cohort cases (**A**) and Illumina HiSeq cohort cases (**B**). Diamonds indicate centroids of each subtype (plotted in two-dimensional space) and values (d) next to the grey dashed line, which indicates the Euclidean distance between the subtype centroids.

Receiver operating characteristic (ROC) curve analysis revealed perfect test results and the ability to discriminate between subtypes (testing subtype versus other two subtypes). Comparable results between both classifiers using an expression array and sequencing data were obtained (area under the curve—AUC > 0.9, Figure 2). The discrimination of the PN subtype showed the best test parameters compared with MES and CL subtypes. To visualize the distance between the cases of different subtypes, we applied multidimensional scaling (MDS) analysis to the data (including the set of all 20 selected genes) and calculated the Euclidean distance between centroids of the clusters. Analysis revealed PN subtype specimens to be more distally located in relation to the MES and CL subtypes in both datasets (see Figure 3).

**Figure 4:**
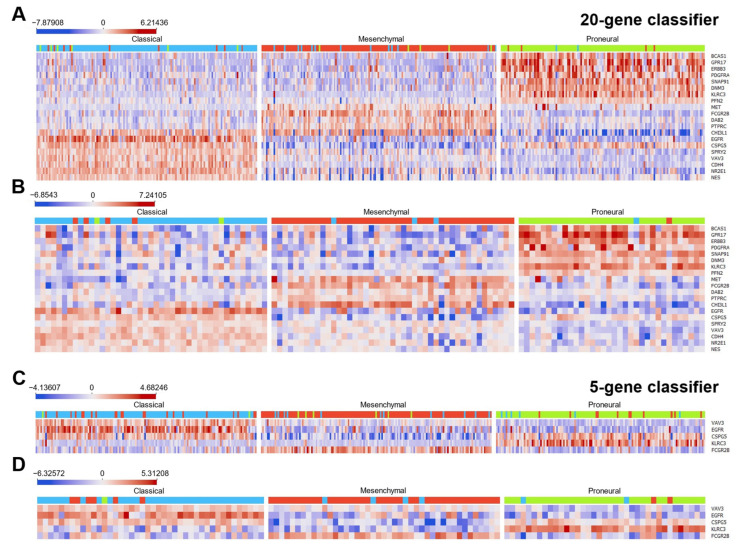
The heatmaps of selected glioblastoma classification genes panels. Blue, red, and green indicators above each heatmap show predicted classical, mesenchymal, and proneural subtypes, respectively. Each color discrepancy in the indicators means sample misclassification with reference to the Verhaak subtype. Data in the heat map are ordered after hierarchical bi-clustering. Parts (**A**,**B**), accordingly, represent data from Affymetrix HT and Illumina HiSeq cohorts applying a 20-gene classifier and parts (**C**,**D**) applying a five-gene classification of the same cohorts, respectively.

The match of the classified cases to the original specimens’ subtype assignment (according to Verhaak) is shown in the heatmap (see Figure 4).

The gene selection approach allows one to select genes with significant expression differences between molecular subtypes (*p* < 0.001, ANOVA). *BCAS1, GPR17, ERBB3, PDGFRA, SNAP91, DNM3, KLRC3*, and *PFN2* gene expressions were significantly increased in PN compared to MES and CL cases. *MET, FCGR2B, DAB2,* and *PTPRC* levels were significantly increased in MES cases compared with the other two subtypes, while *EGFR, SPRY2*, *VAV3, CDH4, NR2E1,* and *NES* expressions were significantly increased in CL cases. *CHI3L1* and *CSPG5* expression was significantly decreased in PN and MES subtypes, respectively (see Figure 4). The pattern of the heat map was repeated between datasets, and, for instance, the genes that were upregulated in the PN subtype in the Affymetrix HT expression array cohort were similarly upregulated in the Illumina HiSeq dataset.

### 2.4. The Construction of the Gene Expression System Applying Ordinary qPCR for Glioblastoma Subtyping

To deliver a functional glioblastoma molecular classification tool, we also developed technical conditions for the analysis of selected genes’ expression by applying qPCR. Since different target sequences selected for amplification of the same gene may result in different gene expression measurements, we designed an expression analysis system based on microarray probe sequences. We used microarray probe sequences to construct qPCR primers that fully or partly covered the probe sequences. All the primers were designed to anneal at 60 °C to enable expression examination of all genes in a single plate. Primer sequences and qPCR conditions are provided in Table 2. Three endogenous controls previously reported to be suitable for glioma expression research were applied for this study. It should be noted that both the five-gene and 20-gene classification models are designed by applying the indicated housekeeping genes.

Gene expression measurements were performed in 56 human glioblastoma specimens. The dataset was named the LUHS (Lithuanian University of Health Sciences) cohort. The molecular and demographical characteristics of patients are shown in Table 3. Patients’ median age was 57.7 years (range 31–80 years) in the LUHS cohort, 59.43 years (range 19–89 years) in the Affymetrix HG cohort, and 60.5 years (range 21–89 years) in the Illumina Hiseq cohort. The median overall survival time after diagnosis was 13.96 months (range 1.51 to 65.41 months), 10.42 months (range 1.21 to 127.56 months), and 9.4 months (range 1.21 to 47.6 months), respectively, in the LUHS, Affymetrix HG, and Illumina Hiseq cohorts.

### 2.5. Subtype Analysis in LUHS Cohort

**Figure 5:**
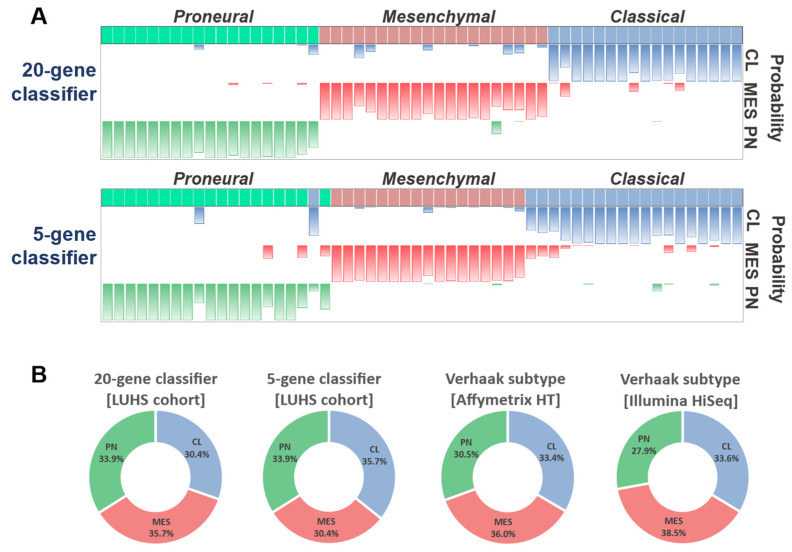
(**A**) LUHS cohort classification comparison between 20-gene and five-gene classification models. Green, red, and blue colors indicate PN, MES, and CL subtypes, correspondingly. The height of each color bar represents the calculated subtype probability for each sample. (**B**) Comparison of subtype proportions in the LUHS cohort was classified by applying 20-gene and five-gene models and TCGA cohorts classified by Verhaark.

Gene expression data were obtained as CT values and were normalized to the geometric mean of three endogenous control genes’ (*ACTB, GAPDH,* and *YWHAZ*) CT values (ΔCT). Data of each analyzed gene were normalized (centered) based on the cohort average and x-x^−^ (where x = data value; x^−^ = mean of a dataset) was included. Then LUHS cohort was tested by applying both 20-gene and five-gene classification models. The calculated molecular subtype matched in 52 specimens out of 56 (92.86%) when comparing the 20-gene and five-gene models’ outputs. The highest match was in the PN group, where 18 out of 19 (94.7%) specimens were identically assigned. MES and CL groups demonstrated a slightly lower match—17 out of 20 (85%) were identically assigned in both cases (Figure 5A).

Next, we performed a subtype proportion comparison after classifying the LUHS cohort by both models. A Z-score test was used to compare subtype proportions between cohorts. Analysis revealed no difference between LUHS cohort subtypes, calculated applying 20-gene or five-gene models, compared with the Affymetrix HG and Illumina HiSeq datasets classified according to the Verhaak system (*p* > 0.05). The proportions of each cohort are shown in Figure 5B.

**Figure 6:**
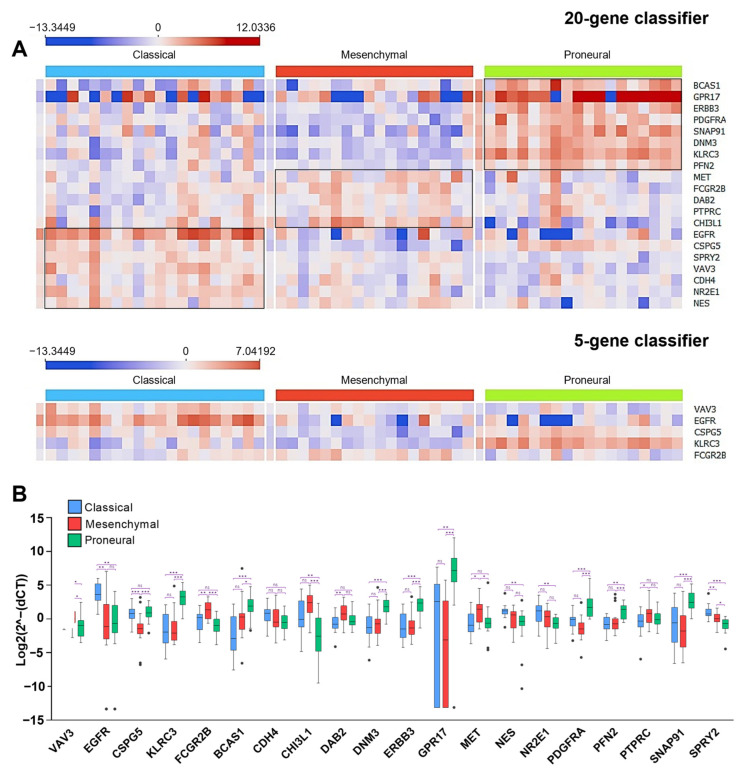
(**A**) Selected gene expression profiles between different subtypes in the LUHS cohort. Specimens are classified according to the 20-gene model (upper) and five-gene model (below). (**B**) Box-plot demonstrates gene expression differences between subtypes in the LUHS cohort (classified based on the 20-gene model). Asterisk indicates significance level: *—*p* < 0.05, **—*p* < 0.01, ***—*p* < 0.001.

We visualized the LUHS cohort gene expression profile in a heat map to elucidate if the same genes that were up- or down-regulated in the TCGA dataset subtypes were similarly aberrant in the LUHS dataset (Figure 6A). The analysis demonstrated that the same genes that were significantly upregulated, for instance in the PN subtype in the TCGA cohorts (*BCAS1, GPR17, ERBB3, PDGFRA, SNAP91, DNM3, KLRC3*, and *PFN2*), were also upregulated in PN samples of the LUHS cohort that was classified by applying the 20-gene classifier (see Figure 6B). Similar data were obtained by comparing MES and CL-specific genes (Figure 4 and Figure 6). It is worth mentioning that the expression levels of all screened-out genes (applying the design described above) were significantly deregulated between subtypes (Figure 6B).

**Figure 7:**
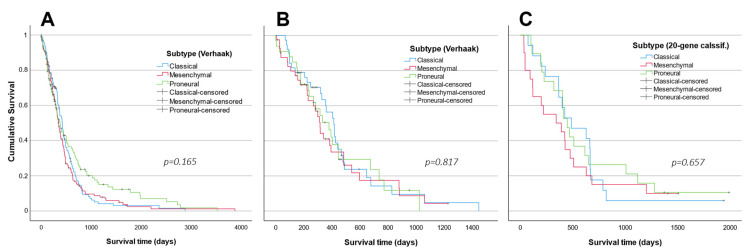
Kaplan–Meier survival curves of (**A**) the Affymetrix HG and (**B**) the Illumina HiSeq patients classified into three groups according to the Verhaak system and (**C**) the LUHS cohort survival curves classified into three groups applying the developed 20-gene classifier.

Survival analysis did not reveal a significant difference between subtypes in any analyzed cohort (*p* > 0.05) (Figure 7). The log-rank test of the TCGA dataset (Affymetrics HT) showed a tendency towards better survival prognosis for PN patients (χ^2^ = 3.601, df = 2, *p* = 0.165), as well as PN, MES and CL survival means of 669, 484, and 503 days, respectively (Figure 7A). Neither the Illumina HiSeq cohort classified according to the original Verhaak system, nor the LUHS cohort classified using the 20-gene classifier, revealed such a tendency (Illumina HiSeq: χ^2^ = 0.404, df = 2, *p* = 0.817; LUHS cohort 20-gene classified: χ^2^ = 0.84; df = 2, *p* = 0.657) (Figure 7B,C). The shortest mean survival estimates were in the MES subtype patients in all cohorts.

## 3. Discussion

Most glioblastoma studies incorporate a layer of tumor molecular subtyping based on the classification system instituted by Verhaak et al., 2010 [6]. Originally, Verhaak et al. classified glioblastomas into four subtypes based on the expression of 840 genes [6]. However, the large number of genes used in the original classifier encourages scientists to optimize the number of features to save expenses and time and to make the molecular classification more applicable. Even though the Verhaak subtyping system has become the most acceptable model, researchers are still applying different methods and markers to assign subtypes to the newly analyzed glioblastoma cohort. Several glioblastoma molecular classification models have been suggested since the Verhaak four-group subtype system was proposed [7,8,10,11,12,20,21,22,23,24]. Nevertheless, most of them provide lists of informative subtyping molecules rather than the classification tool or algorithm itself [7,8,10,11,12,23,24]. Some of the provided glioblastoma classification schemes reclassify tumors in novel subtypes/groups/clusters. However, such a reclassification is incomparable among the studies that were previously published [7,8,24]. During the past decade, two GBM classification tools, based on the Verhaak four-group classification, were proposed in 2014 [20] and 2016 [21], which use 121 and 48 molecules for GBM classification, respectively. Relatively large numbers of genes do not make these tools highly attractive, since the analysis of at least 48 targets still needs high throughput approach. Moreover, recently, few studies have demonstrated that specimens of the neural subtype are samples with high content of normal tissue rather than separate glioblastoma subtypes [11,12,25], indicating the need for a standardized three-group glioblastoma molecular classification tool. Thus, with this work, we suggest a simple and cost-efficient three-class glioblastoma molecular classification tool based on mRNA analysis of five to 20 genes. We hope that this model will encourage researchers and physicians to use the suggested glioblastoma subtyping model more frequently in the future.

The overall accuracy of the proposed glioblastoma subtyping models (five-gene and 20-gene) varied between ~86% and 91%. The original Verhaak classifier was developed by applying silhouette width filtering when selecting only the “core samples”, which then were used for model construction [6]. Thus, achieving absolute classification accuracy is not a feasible task, even when incorporating all 840 features for the classification. Current classification models are comparable with Crisman et al.’s published four-subtype 48-gene model, where accuracy varied between 81.48% and 91.88%, depending on the dataset used [21]. Madurga et al. reduced the classification genes list to 20 genes that showed 89–90% classification accuracy [12]. Pal et al. defined a four-group glioblastoma molecular classifier based on 121 isoform-level gene signatures and received 92% accuracy [20]. Taking this all together, the proposed classification models show similar classification accuracy to those published previously. Nevertheless, the numbers of the features (genes) are considerably smaller. As might be expected, the five-gene model revealed slightly lower classification accuracy values (85.9–87.7%), as compared to the 20-gene model (90.7–91%). Nevertheless, considering that the five-gene model requires four times fewer resources and time, the five-gene model is an excellent choice to classify glioblastoma cohorts in a cost-effective way. Differently from the previously proposed models, current tools provide not only qualitative information about the calculated molecular subtype, but they also provide qualitative information—the probability of the predicted subtype.

The current study, in addition to a list of the genes and the classification model, also provides a user-friendly tool with all instructions for glioblastoma cohort subtype identification. Moreover, the current study provides technical information on optimized and validated qPCR conditions that enable data reproducibility and comparability between the researchers if they follow the described protocol. Different target sequences selected for amplification of the same gene may result in different expression levels. Thus, here we are providing designed and tested primers and optimized qPCR conditions to maximize the reproducibility of the data. Thus, we hope that the proposed glioblastoma classification model with basic information for target analysis in a wet-lab stage will encourage researchers and physicians to use the suggested system more frequently in the future.

## 4. Materials and Methods

### 4.1. TCGA Gene Expression Data Processing

The Cancer Genome Atlas (TCGA) coordination center data [27] were used for developing the classifier. Gene expression data of GBM patients with known *IDH* status, survival data, and Verhaak subtype were collected from the UCSC Xena [28] and the GlioVis [29] data portals. We used level 3 interpreted data of gene expression estimates given in log space, which were mapped onto the human genome coordinates using the UCSC Xena HUGO probeMap [28]. The analysis encompassed three public datasets generated by three gene expression array platforms: the Affymetrix HT Human Genome U133a microarray platform (*n* = 539), the Agilent 244K custom gene expression G4502A microarray platform (*n* = 585), and the RNA sequencing platform Illumina HiSeq 2000 RNA Sequencing (*n* = 172). The cases for the analysis were selected on the basis of four criteria: the subtype of a sample according to Verhaak was specified; the sample type was a primary tumor (recurrent or secondary tumors were eliminated); the case had information about all targets selected for the analysis; and the tumor was not treated prior to resection. After data filtering, based on the above-listed criteria, 505 cases from the Affymetrix HG-U133a, 505 cases from the Agilent G4502, and 162 cases from the Illumina HiSeq datasets were selected for further steps, respectively. Cases that were assigned to the neural subtype were removed from the datasets. The final gene expression microarray dataset consisted of 419 cases, and the RNA sequencing dataset consisted of 122 cases. Because the development of models incorporated different datasets, the data were normalized by applying a standard score: $z=\left(x-u\right)/\sigma$.

### 4.2. Patient Samples

A total of 56 human glioblastoma specimens diagnosed according to the guidelines of WHO, classification 2016, 4th edition (update 3), were used for gene expression analysis. The surgical resections of GBM tumors were performed at the Department of Neurosurgery, Hospital of Lithuanian University of Health Sciences, from September 2017 to June 2021. Tumorous tissues of glioblastoma patients were collected, stored, and analyzed following written informed consent after approval by the Kaunas region Ethics Committee for Biomedical Research (permission code: P2-9/2003). The study was performed following the Lithuanian regulations, alongside the principles of the Helsinki and Taipei Declarations. All tissue samples after surgical resection were snap-frozen and stored in liquid nitrogen. Clinical data (gender and age at the time of resection,) as well as survival data, were collected for each patient. The overall patient survival rates were calculated from the date of tumor resection to the date of patient death or database closure (15 May 2022). None of the patients had received chemotherapy or radiotherapy prior to surgery. The demographic and molecular characteristics of patients are shown in Table 3.

### 4.3. RNA Isolation and qRT-PCR

GBM tumor specimens were homogenized by applying cryogenic grinding with liquid nitrogen, and total RNA from pulverized tissue was extracted using the TRIzol reagent (Invitrogen, Vilnius, Lithuania, cat. #: 15596026). In total, 2 µg of total RNA was used for cDNA synthesis by applying the High-Capacity cDNA Reverse Transcription Kit (Applied Biosystems, Bleiswijk, The Netherlands, cat. #: 4374966). Selected targets’ mRNA expression was investigated by applying quantitative RT-PCR SYBR Green I assay, in three replicates, on a 7500 Fast Real-time PCR detection system (Applied Biosystems, Foster City, CA, USA). The PCR reaction consisted of 3 μL (15 ng when calculating from RNA used for cDNA synthesis) of cDNA, 6 µL of 2x Power SYBR™ Green PCR Master Mix (Applied Biosystems, Bleiswijk, The Netherlands, cat. #: 4368702), 0.29–0.67 µM of primer, and nuclease-free water. For detailed information on primer sequences, amplicons, primer amounts per reaction, as well as PCR cycling conditions, see Table 2. Relative quantification method—ΔCT (when normalized to reference genes) was used for data normalization. Data were normalized according to the geometric mean of the CT estimate of three reference genes (*ACTB*, *GAPDH*, and *YWHAZ*). The final values were used as $2^{-\Delta \mathrm{CT}}$ (fold change) calculations.

### 4.4. Data Analysis

Differences across two independent groups were analyzed by applying a *t*-test. A chi-square test was used for categorical data analysis. Survival analysis was performed by applying the Kaplan–Meier curve method, and a log-rank test was used to compare the difference in survival curves across groups. To show the reliability of the survival estimate, the confidence interval (CI), with a 95% confidence level, was presented. The level of significance was *p* < 0.05. Data visualization, target selection, model construction, and testing were performed using the machine learning and data visualization toolkit “Orange” (ver.3.32, University of Ljubljana, Ljubljana, Slovenia). IBM SPSS Statistics (V27.0.1.0, New York, NY, USA) and GraphPad Prism (V6.01, GraphPad Software, Inc., San Diego, CA, USA) software were used for statistical analysis and data visualization.

## Figures and Tables

**Table 1:** The comparison of different models’ performance during the construction of the classifiers and testing stages.

**Classifier Development**						
Dataset: **Agilent G4502**	Logistic regression			SVM		
	**20-gene model**	5 top ranked genes model	**5 selected genes model**	20-gene model	5 top ranked genes model	5 selected genes model
Classification accuracy	0.948	0.877	0.937	0.948	0.877	0.932
Area under ROC curve (AUC)	0.995	0.888	0.995	0.994	0.888	0.994
**Classifier testing**						
Dataset: **Affymetrix HT**						
Classification accuracy	0.907	0.833	0.859	0.914	0.835	0.859
Area under ROC curve (AUC)	0.967	0.933	0.946	0.986	0.946	0.961
Dataset: **Illumina HiSeq 2000**						
Classification accuracy	0.91	0.885	0.877	0.893	0.869	0.868
Area under ROC curve (AUC)	0.998	0.991	0.992	0.987	0.977	0.985

**Table 2:** Primer sequences and qPCR conditions.

		Gene Name	Forward Primer 5′ --> 3′	Revers Primer 5′ --> 3′	Amplicon Lenght, bp	Primer Amount µM	Annealing Temp., °C
20-gene classifier	5-gene class.	*CSPG5*	CTCTACCTGCTCAAGACGGA	GCACTAGGATCATCATTTGGGT	133	0.33	62
		*EGFR*	GGACCAGACAACTGTATCCA	AAGATTTATTAGGACCCGTAGGTG	172	0.67	60
		*FCGR2B*	CTGTGCTTTCTGAGTGGCTG	TGACTGTGGTTTGCTTGTGG	189	0.29	62
		*KLRC3*	ATATGACTGCCAAGGTTTACTG	CTCTTCCCAAGTTCTTCTTTCC	246	0.29	60
		*VAV3*	CTCAAACTACCAGAGAAACGGAC	ATCTCCTTTCAGAAGTTCAACGG	176	0.33	60
		*BCAS1*	AGACAAATGACATCAGACTCCA	CTTCTGCTTGTTCATCTCGG	131	0.42	60
		*CDH4*	CCGTCCCAGAATATGTTCAC	GCCATAGTTGAGATTTCCTTCC	137	0.42	58
		*CHI3L1*	GTCTCAAACAGGCTTTGTGG	GTAGATGATGTGGGTACAGAGG	153	0.42	60
		*DAB2*	CAGTTGAGAATGGGAGTGAGG	GTGGGAAAGAAGTTGAGATTGG	240	0.33	54
		*DNM3*	TCCTCAAGGTCTGAGAACCA	GTCCTTCTTCCCATCTATGTCC	159	0.42	60
		*ERBB3*	ATGCTGAGAACCAATACCAGAC	CAAACTTCCCATCGTAGACCT	255	0.42	60
		*GPR17*	AGCAGCTAGAGGATGTCCA	TGGAGTCAGAGCCTGAGAG	87	0.29	60
		*MET*	CACTGCTTTAATAGGACACTTCTG	AGGTGGATATAGATGTTAAGAGGAC	147	0.42	60
		*NES*	GTTGGAACAGAGGTTGGAGG	AAAGCTGAGGGAAGTCTTGG	173	0.42	60
		*NR2E1*	TCAAGTGGGCTAAGAGTGTG	ACCGTTCATGCCAGATACAG	160	0.29	60
		*PDGFRA*	ACAACCTCTACACCACACTG	ATGATCTCGTAGACTTCACTGG	180	0.29	60
		*PFN2*	GTTTCTTTACCAACGGTTTGAC	CATGACTATAACCAATGCTCTACC	169	0.42	60
		*PTPRC*	TAAGACAACAGTGGAGAAAGGAC	CAAATGCCAAGAGTTTAAGCCA	96	0.42	60
		*SNAP91*	CCCAGTCAGCACTTCTAAACC	CAGCCAAAGAATCCTCTCCC	154	0.42	60
		*SPRY2*	GGAAGTTGGTCTAAAGCAGAGG	CACATCTGAACTCCGTGATCG	137	0.29	60
Endogenous contr.		*ACTB*	AGAGCTACGAGCTGCCTGAC	AGCACTGTGTTGGCGTACAG	184	0.083	60
		*GAPDH*	TCAAGATCATCAGCAATGCCT	CATGAGTCCTTCCACGATACC	94	0.42	60
		*YWHAZ*	CCGTTACTTGGCTGAGGTTG	TGCTTGTTGTGACTGATCGAC	67	0.42	62

**Table 3:** Summary of patient molecular and demographical characteristics.

Features	LUHS Cohort *n* = 56	Affymetrix HG-U133a *n* = 419	Illumina HiSeq 2000 *n* = 122
Gender			
Female	29 (51.8%)	166 (39.6%)	47 (38.5%)
Male	27 (48.2%)	253 (60.4%)	75 (61.5%)
Age (years)	mean 58.66	mean 58.2	mean 60
≤60	30 (53.6%)	216 (51.6%)	53 (53.4%)
>60	26 (46.4%)	203 (48.4%)	69 (56.6%)
Survival (months)	mean 17.78	mean 14.51	mean 11.3
≤12	20 (35.7%)	242 (57.7%)	72 (59%)
>12	36 (64.3%)	177 (42.3%)	50 (41%)
IDH1 mutation		Unexplored *n* = 107	Unexplored *n* = 13
Wild-type	50 (89.3%)	286 (91.6%)	101 (92.7%)
Mutant	6 (10.7%)	26 (8.4%)	8 (7.3%)
MGMT methylation		Unexplored *n* = 146	Unexplored *n* = 34
Unmeth	28 (50%)	137 (50.2%)	46 (52.3%)
Meth	28 (50%)	136 (49.8%)	42 (47.7%)

## Data Availability

Data is available from the corresponding author upon reasonable request via email.

## Supplementary Materials

The following supporting information can be downloaded at: https://www.mdpi.com/article/10.3390/ijms232415875/s1.
