# Supplementary material for: Glioblastoma Molecular Classification Tool Based on mRNA Analysis: From Wet-Lab to Subtype
Source: Int J Mol Sci. 2022 Dec 14;23(24):15875. doi: 10.3390/ijms232415875 (PMC9784712; doi:10.3390/ijms232415875)
Supplement: Supplementary file 1 [file ijms-23-15875-s001.zip › ijms-2056984-supplementary.pdf]

Table S1. Overlapping signature genes per different datasets and their expression correlation between platforms

No.	***Signatures proposed elsewhere*** • **Verhaak et al, 2010;** • **Phillips et al, 2006;** • **Wang et al, 2017;** • **Sandmann et al, 2015**	***Genes level measurements available at:*** • **Agilent G4502** • **Affymetrix HG-U133a** • **Illumina HiSeq**	**Correlation between platforms (Spearmans r)**	**FDR**	**Selected**
1	ABL1	ABL1	0,502	4,15E-32	No
2	ANGPTL4	ANGPTL4	0,898	5,2E-176	Yes
3	ATP6V1G2	ATP6V1G2	0,714	3,23E-77	Yes
4	BCAS1	BCAS1	0,879	3,4E-159	Yes
5	BOP1	BOP1	0,604	1,38E-49	No
6	CASP1	CASP1	0,82	2,2E-120	Yes
7	CASP4	CASP4	0,696	5,87E-72	Yes
8	CASP5	CASP5	0,499	9,56E-32	No
9	CASP8	CASP8	0,442	2,12E-24	No
10	CCR5	CCR5	0,627	1,91E-54	No
11	CD4	CD4	0,637	1,01E-56	No
12	CDH4	CDH4	0,821	5,4E-121	Yes
13	CDK6	CDK6	0,61	9,25E-51	No
14	CENPK				
15	CHI3L1	CHI3L1	0,713	5,02E-77	Yes
16	COL4A1	COL4A1	0,817	8E-119	Yes
17	COL4A2	COL4A2	0,801	5,9E-111	Yes
18	CSDC2	CSDC2	0,639	3,9E-57	No
19	CSPG5	CSPG5	0,691	2,64E-70	Yes
20	DAB2	DAB2	0,798	1,8E-109	Yes
21	DLL3	DLL3	0,861	1,5E-145	Yes
22	DNM3	DNM3	0,74	9,09E-86	Yes
23	DNMT1	DNMT1	0,747	1,75E-88	Yes
24	DTL	DTL	0,807	1,2E-113	Yes
25	E2F7				
26	EGFR	EGFR	0,865	2,9E-148	Yes
27	PLA2G5	PLA2G5	0,75	2,52E-89	Yes
28	ERBB3	ERBB3	0,699	8,92E-73	Yes
29	FAM21C				
30	FBXO3	FBXO3	0,567	2,04E-42	No
31	FCGR2B	FCGR2B	0,849	2,9E-137	Yes
32	FERMT1	FERMT1	0,86	2,6E-145	Yes
33	FGFR3	FGFR3	0,85	6,5E-138	Yes
34	FOSL2	FOSL2	0,791	3,9E-106	Yes
35	GABBR1	GABBR1	0,693	7,87E-71	Yes
36	GABRB2	GABRB2	0,135	0,003774	No
37	GALNT13				
38	GPR17	GPR17	0,816	1,4E-118	Yes
39	HMMR	HMMR	0,64	2,34E-57	No
40	IL4R	IL4R	0,779	6,1E-101	Yes
41	IQGAP3				

Table S1. Overlapping signature genes per different datasets and their expression correlation between platforms

42	KLRC3	KLRC3	0,824	1,1E-122	Yes
43	LIF	LIF	0,798	1,7E-109	Yes
44	MBP	MBP	0,842	6,9E-133	Yes
45	MET	MET	0,708	1,76E-75	Yes
46	MYL9	MYL9	0,768	2,17E-96	Yes
47	NCAM1	NCAM1	0,537	2,06E-37	No
48	NDRG2	NDRG2	0,799	7,9E-110	Yes
49	NES	NES	0,691	2,39E-70	Yes
50	NKX2.2	NKX2.2	0,729	6,52E-82	Yes
51	NR2E1	NR2E1	0,774	6,9E-99	Yes
52	OLIG2	OLIG2	0,72	4,54E-79	Yes
53	OPHN1				
54	PDGFA	PDGFA	0,808	3,2E-114	Yes
55	PDGFRA	PDGFRA	0,885	2,3E-164	Yes
56	PDLIM4	PDLIM4	0,896	7,4E-175	Yes
57	PDPN	PDPN	0,814	3,1E-117	Yes
58	PFN2	PFN2	0,819	1,1E-119	Yes
59	PTPRC	PTPRC	0,775	3,5E-99	Yes
60	RASL10A	RASL10A	0,823	4,1E-122	Yes
61	RELB	RELB	0,656	4,99E-61	Yes
62	SCG3	SCG3	0,779	4,8E-101	Yes
63	SERPINE1	SERPINE1	0,853	3,1E-140	Yes
64	SNAP91	SNAP91	0,771	1,35E-97	Yes
65	SNCG	SNCG	0,45	2,57E-25	No
66	SOX2	SOX2	0,679	5,59E-67	Yes
67	SOX8				
68	SPOCD1				
69	SPRY2	SPRY2	0,816	4,1E-118	Yes
70	SRRM2	SRRM2	0,689	7,42E-70	Yes
71	TAGLN	TAGLN	0,831	2,2E-126	Yes
72	TIMP1	TIMP1	0,874	1,9E-155	Yes
73	TLR2	TLR2	0,777	5,9E-100	Yes
74	TLR4	TLR4	0,625	5,49E-54	No
75	TOP1	TOP1	0,525	1,57E-35	No
76	TRADD	TRADD	0,721	1,83E-79	Yes
77	VAV3	VAV3	0,872	2,7E-154	Yes
	**Total: 77**	**Total: 69**			**Total: 54**
